# Treatment of diffuse sclerosing osteomyelitis of the jaw with denosumab shows remarkable results—A report of two cases

**DOI:** 10.1002/ccr3.1894

**Published:** 2018-10-26

**Authors:** Fredrik Hallmer, Mikael Korduner, Anne Møystad, Tore Bjørnland

**Affiliations:** ^1^ Department of Oral and Maxillofacial Surgery Skåne University Hospital Lund Sweden; ^2^ Faculty of Odontology Malmö University Malmö Sweden; ^3^ Faculty of Dentistry, Institute of Clinical Odontology University of Oslo Oslo Norway; ^4^ Faculty of Dentistry, Department of Oral Surgery and Oral Medicine University of Oslo Oslo Norway

**Keywords:** denosumab, mandible, osteomyelitis, treatment

## Abstract

Denosumab may play a central role in the treatment of diffuse sclerosing osteomyelitis of the mandible. This report describes two patients who had been treated unsuccessfully with antibiotics and steroids for several years. After denosumab treatment, both patients became pain‐free and the radiological examination showed less severe osteomyelitis.

## 1 INTRODUCTION

Diffuse sclerosing osteomyelitis (DSO) of the mandible is a rare chronic condition, the cause of which is poorly understood. It is often initiated by an oral infection but later changes to a sterile chronic osteomyelitis.^1^, ^2^

Severe jaw pain, occurring irregularly, is a typical symptom. Clinically, it is characterized by mandibular swelling caused by an inflammation of the bone marrow, involving the cortical plates and often periosteal tissues.^3^ The radiological appearance of DSO includes sclerosis, partial osteolysis and periosteal bone formation, widening of the lamina dura, and the diffuse border of the mandibular canal.^4^, ^5^

Current treatment protocols include antimicrobial therapy, hyperbaric oxygen, steroid or analgesic medication, and surgical debridement.^6^, ^7^ However, these treatment options often show poor outcomes. Several reports have been published with promising results concerning the treatment of DSO with bisphosphonates.^1^, ^8^, ^9^

Bisphosphonate inhibits bone resorption, bone turnover, and renewal through inhibition of the osteoclasts.^10^, ^11^ As with bisphosphonate, denosumab, also inhibits bone resorption and is a monoclonal antibody against the RANKL, the ligand of the receptor activator of nuclear factor‐*K*B. Denosumab inhibits RANKL from activating its receptor, RANK, on the surface of osteoclasts and their precursors. Prevention of RANKL‐RANK interaction by denosumab inhibits osteoclast differentiation, function, and survival, thereby decreasing bone resorption.^12^

We report two cases where denosumab was used to treat DSO where other treatment options were unsuccessful, and we use these cases to discuss limitations of the treatment options.

## 2 CASES PRESENTATION

### 2.1 Clinical case 1

A 14‐year‐old girl, who in June 2010 had a primary molar (tooth 75) extracted on orthodontic indications, developed DSO. After a prolonged healing period involving pain and swelling, the patient was referred to a specialist in oral and maxillofacial surgery at Växjö County Hospital. The patient was diagnosed with acute osteomyelitis in November 2010 with swelling, pain, radiographic symptoms, and a biopsy that showed osteomyelitis with periosteal activity.

The acute osteomyelitis was treated with clindamycin and six months later the clinical and radiographic signs showed diffuse sclerosing osteomyelitis, without other signs of odontogenic infection, temporomandibular disorder, or impacted wisdom teeth in need of extraction that could explain her symptoms. The patient was then referred to the Department of Oral and Maxillofacial Surgery, Skåne University Hospital, Lund, for further diagnosis and treatment.

**Figure 1:**
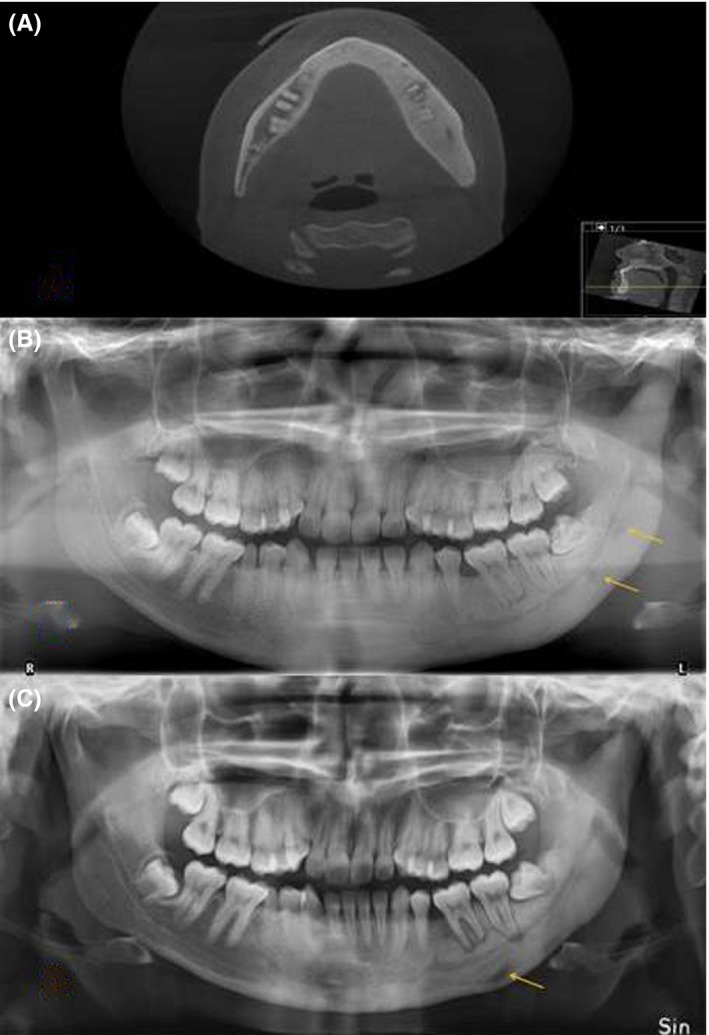
Radiological examinations of a 21‐year‐old woman with diffuse sclerosing osteomyelitis of the left side of the mandible treated with cortisone and analgesics for five years. A, CT before the treatment with denosumab, revealing sclerosis of the entire left side of the mandible, crossing the midline, and some thickening of the mandible and widening of lamina dura around teeth 36, 37, and 38 as typical signs of diffuse sclerosing osteomyelitis. B, Orthopantomogram before the treatment with denosumab, showing sclerosis of the left side of the mandible with areas of radiopaque areas, and some bone apposition. C, Orthopantomogram after 20 mo with denosumab treatment showing more radiolucency around teeth 36, 37, and 38, indicating bone resorption but somewhat less sclerosis. The teeth 36, 37, and 38 had been vital throughout the treatment period

She was then treated for five years with corticosteroid and NSAID but with unsatisfactory results without pain relief. Therefore, other treatment options were discussed and due to the shorter half‐life of denosumab compared with bisphosphonate, denosumab was considered. Before treatment with denosumab (Figure 1A,B), she was informed of the risk of medication‐related osteonecrosis of the jaw (MRONJ) that could be initiated by denosumab.^13^, ^14^

Subcutaneously, 120 mg denosumab (February 2014) was administered. Three days after the injection, she was in completely pain‐free and in need of no other pain relief medication. During the first three months, she was given 120 mg every month. After completion of the initial treatment with denosumab, the pain then started again six months later but with less intensity and 120 mg denosumab (May 2015) was given. Three or four days after the injection, she was pain‐free and this lasted another five months (October 2015), when the latest injection of denosumab was given with the same successful results as before (Figure 1C).

### 2.2 Clinical case 2

A 71‐year‐old woman diagnosed with DSO had been treated with analgesics (Diclofenac 50 mg x 3), Corticosteroids and antibiotics (Clindamycin 300 mg x 3), in periods between August 2014 and August 2016 but with poor pain relief. Cone‐beam computer scan revealed radiopaque areas at the left corpus and anterior part of the mandible as well as periosteal bone formation. The diagnosis DSO was confirmed with bone biopsy and histological analysis. Clinical and radiological examination ruled out any odontogenic infection and temporomandibular disorder.

**Figure 2:**
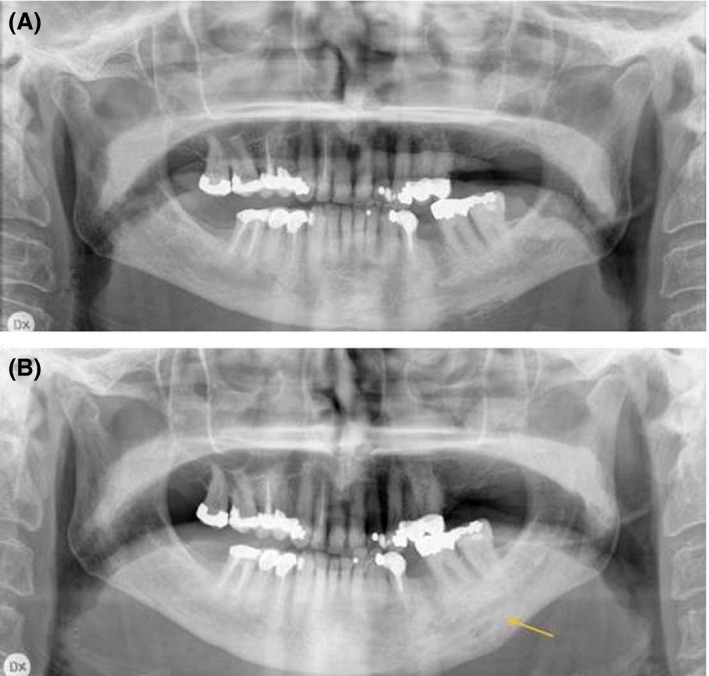
Radiological examinations of a 71‐year‐old woman with diffuse sclerosing osteomyelitis of the left side of the mandible for two years, treated with corticosteroids and clindamycin for two years. A, Orthopantomogram before treatment with denosumab revealing sclerosis, resorption, and periosteal apposition of the left side of the mandible. B, Orthopantomogram taken after 12 mo with denosumab treatment showing less radiolucency and maturation of the bone in the area of periosteal apposition

She classified her symptoms as “pain cannot be worse” and, since no other treatment had been successful and she was unwilling to undergo surgical resection of the jaw, she was offered treatment with denosumab. Before treatment, she was informed of the risk of developing MRONJ (Figure 2A).

In August 2016, the patient was given 60 mg denosumab subcutaneously and five days later she was completely pain‐free and needed no analgesics. During the first three months, she required some analgesics (paracetamol, 1000 mg) at night. After four months (December 2016), the pain started again and a second treatment with 60 mg denosumab was given. Three days after the injection, she was pain‐free and this period without any need of analgesics other than two times (paracetamol, 1000 mg) during a period of four months. After this, the pain started again and a third treatment with 60 mg denosumab (April 2017) was given. Again, three days after the injection, she was completely pain‐free and this period without any need of analgesics, a period of 4 months, lasted until August 2017 when the last follow‐up was recorded (Figure 2B).

## 3 DISCUSSION

There are several treatment options for patients suffering from DSO. The therapies include medical treatment with intravenous antibiotics, oral antibiotics for long‐term use, cortisone, nonsteroid anti‐inflammatory drugs, and surgical treatment with decortication to remove the infected bone or resection. Often, a combination of medical and surgical treatment is required. The medical treatments for DSO need treatment times of several months and often give poor results for pain relief.^1^, ^15^ Surgical treatment with decortication, involving the removal of infected cortical bone and periosteum, aims to increase the blood flow in the area to improve healing.

To the best of our knowledge, this is the first report of treatment of DSO with denosumab where no other antiresorptive treatment has been provided before treatment with denosumab. Our findings show remarkable results with complete pain relief in both patients with DSO, after other established treatments such as analgesics and antibiotics had been given for long periods with poor results.

Similarly successful results have been achieved with bisphosphonate treatment of DSO.^1^, ^8^, ^9^ In the study by Otto et al,^1^ 10 out of 11 patients showed distinct improvement in pain after an infusion of the bisphosphonate ibandronate. One of the patients in that study with recurrence of pain was treated with a subcutaneous injection of 60 mg denosumab, one year after the initial bisphosphonate treatment, resulting in pain relief and reduced inflammatory activity.^4^

There are some theories regarding the pathophysiology of DSO and why antiresorptive drugs might lead to a reduction in pain level. Both Montonen et al^16^ and Otto et al^17^ hypothesized that a disturbance in the RANK/RANKL/OPG system regulates both osteoclasts and osteoblasts, resulting in increased osteoblast activity. This might play a key role in the pathology of DOS. The role of denosumab treatment of giant cell tumor in children resulting in osteosclerosis has also been presented by Kobayashi et al,^18^ with promising results similar to the treatment for DSO.

However, treatment of DSO with denosumab or bisphosphonate has its limitations. The risk of MRONJ must be considered and risk factors contributing to MRONJ must be ruled out before treatment starts.^13^ The pathogenesis of MRONJ is still debated, but recent data suggest that odontogenic infections such as periodontitis might play a crucial role and contribute to the development of the disease.^14^ Therefore, patients with severe periodontal disease or apical periodontitis should have infected teeth extracted before denosumab treatment to eliminate the risk of MRONJ.^15^ Dentoalveolar trauma, such as oral surgery, should also be avoided after treatment with denosumab to avoid the risk of MRONJ. In young patients with DSO, wisdom teeth should be removed before treatment with denosumab.

In case number 1, the periapical lesions around two teeth seemed to increase but sensibility was normal. Therefore, this may have been due to the disease process rather the lack of vitality. Endodontic treatment should therefore not be undertaken at this stage.

The advantages of treatment with denosumab compared with bisphosphonate are a shorter half‐life in bone. The risk of MRONJ after bisphosphonate treatment must be considered for several years while the risk after denosumab treatment can be considered low after six months. In the two patients treated, the denosumab dose seemed to be of less importance since 60 mg was as effective as 120 mg. Thus, this may also lower the risk of developing MRONJ.

In conclusion, the report has highlighted the beneficial effects of denosumab in the treatment of DSO with remarkable pain relief. Further studies, with a control group and long‐time follow‐up are needed.

## CONFLICT OF INTEREST

None declared.

## AUTHOR CONTRIBUTION

FH: involved in project idea, collection of material, and preparation and writing of manuscript. MK: involved in collection of material and preparation and writing of manuscript. AM: involved in evaluation of the material and results and writing of manuscript. TB: involved in evaluation of material and results and preparation and writing of manuscript.
